# Acoustic Characterization Study of Beef Loins Using Ultrasonic Transducers

**DOI:** 10.3390/s23239564

**Published:** 2023-12-01

**Authors:** Antonio Jiménez, Montaña Rufo, Jesús M. Paniagua, Alberto González-Mohino, Teresa Antequera, Trinidad Perez-Palacios

**Affiliations:** 1 Department of Applied Physics, School of Technology, Research Institute of Meat and Meat Product, Universidad de Extremadura, Avenida de la Universidad s/n, 10003 Cáceres, Spain; ajimenez@unex.es (A.J.); mmrufo@unex.es (M.R.);; 2 Department of Food Technology, Faculty of Veterinary, Research Institute of Meat and Meat Product, Universidad de Extremadura, Avenida de la Universidad s/n, 10003 Cáceres, Spain; tantero@unex.es (T.A.); triny@unex.es (T.P.-P.)

**Keywords:** ultrasound parameters, fresh beef loins, ultrasonic transducers, quality parameters

## Abstract

The objective of this study was to non-destructively characterize samples of fresh beef loin by low-intensity ultrasound inspection at various frequencies and to correlate the acoustic parameters of these inspections with quality parameters. In this regard, ultrasonic parameters such as ultrasound pulse velocity (UPV) and variables related to attenuation and frequency components obtained from fast Fourier transform (FFT) were considered. For this, pulsed ultrasonic signal transducers with a frequency of 0.5 and 1.0 MHz were used. Acoustic parameters and those obtained through traditional instrumental analyses (physicochemical and texture) underwent a Pearson correlation analysis. The acoustic determinations revealed numerous significant correlations with the rest of the studied parameters. The results demonstrate that ultrasonic inspection has the ability to characterize samples with a non-destructive nature, and likewise, this methodology can be postulated as a promising predictive tool for determining quality parameters in beef loin samples.

## 1. Introduction

Meat and meat derivatives have great importance for the global economy, where beef meat consumption is constantly increasing and therefore expanding its contribution in the meat industry [1]. Ensuring the quality characteristics of each meat product is an essential goal that the meat industry seeks. Due to this fact, there are many companies dedicated to this sector, mainly because the products obtained are of high quality in the market.

Physicochemical analyses are the conventional quality determinations to obtain a quality profile of meat products. These methodologies have demonstrated their effectiveness [2]; nevertheless, the majority of these analytical techniques cause changes in the meat’s composition or even require the destruction of the sample [3]. Therefore, in recent years, non-destructive techniques have been investigated to reliably evaluate the quality parameters of meat and meat derivatives. These emerging techniques are intended to be alternatives or complements to traditional methods [4].

Low-intensity ultrasound inspection has emerged as a promising and reliable technology for characterizing food quality [5]. With new developments in the areas of basic science related to this method, ultrasonic analysis is expected to be widespread and cost-effective for large-scale meat quality evaluation in the near future [6,7]. Its effectiveness has been proven by assessing different food matrices and—particularly in meat and meat products—with contact [8,9,10] and non-contact methodologies [11,12]. Thus, ultrasonic inspection determines acoustic parameters that allow the characterization of food samples. Although the literature typically focuses on parameters related to wave velocity and attenuation, recent studies have introduced less common acoustic parameters, including frequency components associated with fast Fourier transform (FFT) [8] and attenuation (without visible echo presence in the corresponding A-scan) measured in terms of energy reception time [13]. The contributions of the acoustic characterization of beef meat are scarce, and moreover, only traditional ultrasonic parameters were considered [14,15,16].

In this scenario, the main objective of this proposal is to study the feasibility of non-destructive ultrasonic inspection to characterize beef loin samples using the mentioned acoustic parameters. The application of some of these parameters to the inspection of beef loins is indeed novel. Additionally, the study is conducted using transducers of different frequencies. Finally, a correlation study of the physicochemical characteristics with ultrasonic parameters is intended to be conducted in order to assess their predictive potential.

## 2. Materials and Methods

### 2.1. Samples and Experimental Design

**Figure 1:**
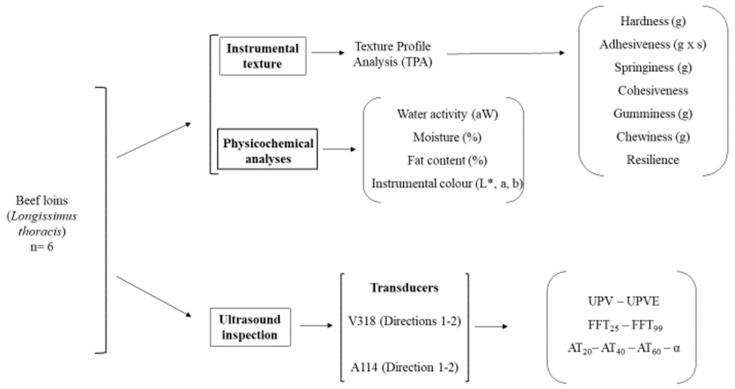
Scheme of the experimental design.

Six fresh beef loins (*Longissimus thoracis*) were purchased from the protected geographical indication (PGI) of Ternera de Extremadura (Cáceres, Spain). These six loins were divided into two parts for the subsequent analyses: one part was used for ultrasound inspection and the other for physicochemical analyses, i.e., twelve parts corresponding to six beef loins. Likewise, the acoustic parameters obtained were correlated with physicochemical ones. Figure 1 displays the scheme of the experimental design.

### 2.2. Physicochemical Analyses

Color and texture determinations, moisture, water activity (aW), and lipid content were analyzed in the beef loins. The beef loins’ instrumental color was assessed using a Minolta CR-300 colorimeter (Konica Minolta, Osaka, Japan) that had been calibrated using a standard white calibration tile. This analysis adhered to the guidelines outlined by the Commission International d’Éclairage (CIE) [17]. The color parameters measured were lightness (L*), redness (a*), and yellowness (b*). Regarding instrumental texture, a TA.XT Plus Texture Analyzer (Stable Micro Systems Ltd., Surrey, UK) was employed. A texture profile analysis (TPA) was performed using a cylindrical probe with a 5 cm diameter. Five cubes (1 cm^3^) were acquired and analyzed for each batch and sample. These cubes were compressed axially to 50% of their original height. The assessed parameters included hardness (g), adhesiveness (g·s), springiness (g), gumminess (g), cohesiveness (dimensionless), and chewiness (g). Moisture content was determined by drying 5 g samples at 102 °C following the official methods of the Association of Official Agricultural Chemists (reference method 935.29) [18]. Water activity (aW) was measured using a water activity measuring instrument (Lab Master-aw, NOVASINA AG, Lachen, Switzerland). Finally, lipid content was assessed in accordance with the method outlined by Folch et al. (1957) [19] employing chloroform:methanol (2:1). All physicochemical determinations were performed in triplicate.

### 2.3. Ultrasound Inspection

**Figure 2:**
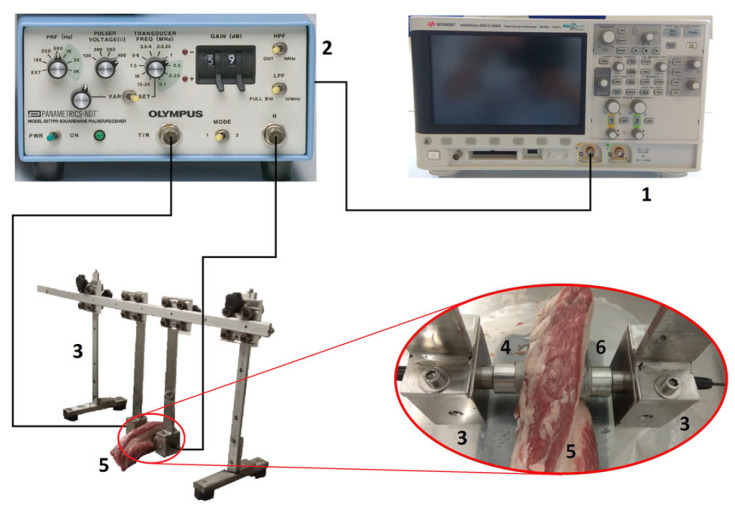
Block diagram for the experimental ultrasonic measurements (1 = oscilloscope, 2 = pulser–receiver, 3 = bespoke metal framework, 4 = transmitter transducer, 5 = beef loin sample, 6 = receiver transducer).

The ultrasound inspection instruments and procedures basically coincide with those described in previous works [9,20] with some modifications; hence, only main methodologies are described. The ultrasound measurements were conducted within a temperature-controlled RIVACOLD RC325-45ED chamber (Montecchio, Italy), maintaining a constant temperature of (7.0 ± 0.2) °C. In this way, variations in the ultrasonic parameters caused by a change in the inspection temperature are avoided [21,22]. The assessment of beef loin samples employed through-transmission mode (TT). Figure 2 displays the setup of the inspection, following and adapting the same procedure as in a previous study in pork loins [9]. Two distinct pairs of transducers were utilized: Panametrics V318-SU and A114-S (Waltham, MA, USA), with a nominal frequency of 0.5 MHz and 1.0 MHz, respectively. The intention behind this was to determine whether the inspection frequency does or does not affect the calculated ultrasonic parameters. Supplementary Material Table S1 provides an overview of the main characteristics of these two pairs of transducers. During the inspection, the transducers were integrated into a customized metal structure that ensured, on one hand, their precise alignment among them and, on the other hand, their isolation from potential signals originating from the metal structure itself through elastic rubber fastenings. Additionally, the transducers were excited at their resonance frequency with a pulse repetition frequency (PRF) of 100 Hz and a voltage of 100 V peak to peak using a Panametrics-NDT Model 5077PR pulser–receiver (Waltham, MA, USA). This model offers negative square wave excitation with a fast pulse rise and fall times (typically < 10 ns, 20 ns max).

**Figure 3:**
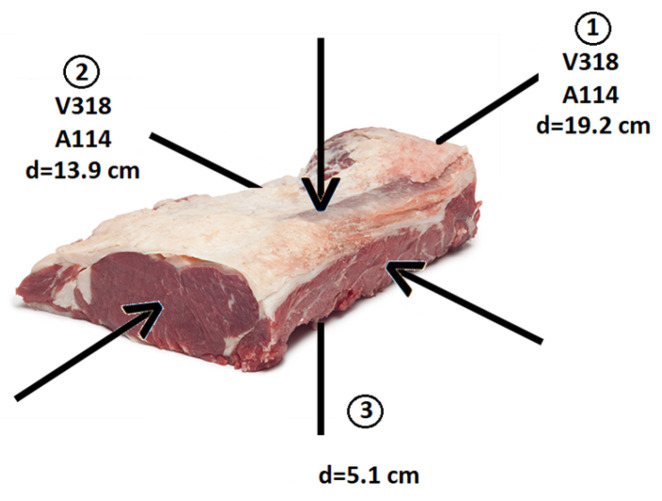
Directions of inspections for both transducers (d = thickness).

The initial selection of the three measurement directions was initially motivated by the fact that the muscle fibers are oriented longitudinally (direction 1) [23], deeming it appropriate to inspect this direction and the other two perpendicular directions. However, the inspection was carried out on each piece in two orthogonal directions, as depicted in Figure 3. This is because the near-field length of transducer A114 exceeds the average length of the loin pieces in direction 3, as can be observed in Table S1. Consequently, this direction was not inspected with any of the transducers. Instead, the distances covered by the ultrasonic waves in the inspections conducted in directions 1 and 2 ensured that the measurements were carried out in the far field (Fraunhofer region). This distance is substantially greater than the length of the near field (Fresnel region) specified in Table S1. The deliberate selection of the far-field region was critical to prevent wave interference phenomena from affecting the evaluation of parameters linked to the attenuation and frequency of the received signal. By working in the far field, any potential interference issues were mitigated, ensuring the accurate assessment of these parameters [24]. Three inspections were conducted for each of the two measurement directions, with an attempt to position the transducers at the center of the surfaces to avoid the presence of visible fat on them.

Furthermore, in the characterization of beef loins, a KEYSIGHT InfiniiVision DSO-X 3032A oscilloscope (Santa Rosa, CA, USA) was employed. Identically, a coupling gel was applied to the surfaces of the beef loins to facilitate the transmission.

**Figure 4:**
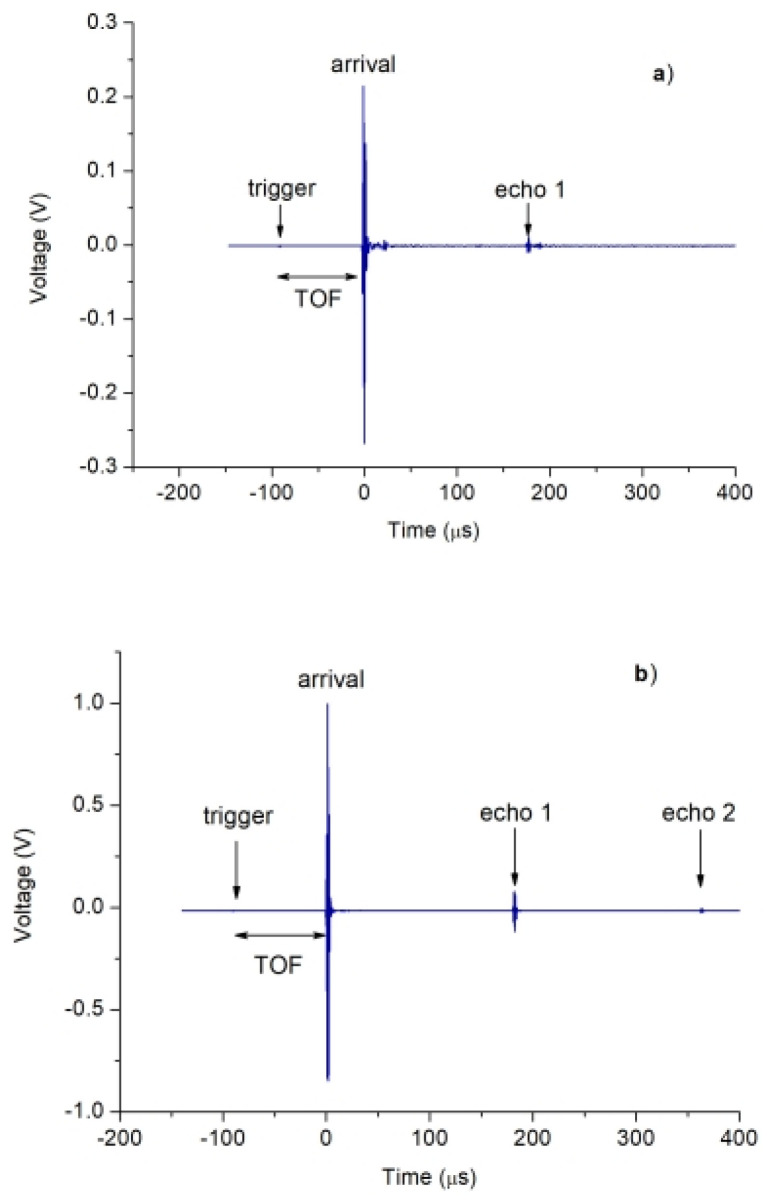
A-scan obtained with the V318 (**a**) and A114-S (**b**) transducers.

Ultrasound parameters were obtained using A-scan data. As an example, Figure 4 displays representative A-scans obtained using V318 and A114 transducers. In both cases, the calculation of the ultrasonic pulse velocity (*UPV*) is based on the time elapsed (time of flight—*TOF*) from signal emission (trigger) to reception (arrival). Since the distance d for each inspection is known, it is possible to determine the *UPV* from *UPV* = d/*TOF*.

Furthermore, in the case of direction 2, the characteristic A-scans obtained also display the arrivals of the first two echoes (for the A114 transducers) or only the first one (for the V318 transducers), originating from successive reflections on the opposing faces of the receiving and transmitting transducers, respectively (see Figure 4). This allows us to assess the velocity *UPVE* by a second method. This method involved performing a least-squares fit to the equation of a straight line, which relates the known distance covered by the ultrasonic signal to the corresponding time of flight for each of the echoes, utilizing for that the time of arrival at the maximum and minimum amplitude values of the respective pulse. Unfortunately, the echoes generated in the inspections conducted in direction 1 with both types of transducers were not visible.

**Figure 5:**
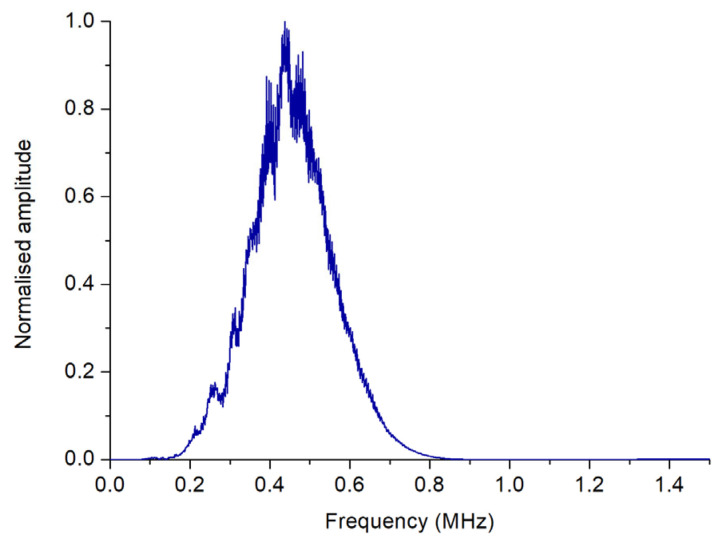
FFT of the A-Scan from Figure 4a.

The frequency parameters were obtained from the fast Fourier transform (FFT) of the received ultrasonic signals, as described in prior studies [9]. Figure 5 shows the FFT of the signals received in the inspection as an example. Using these FFTs, we constructed cumulative frequency periodograms, displaying the 25th and 99th percentiles (*FFT25* and *FFT99*, respectively) of the frequencies present in the received signals. The cumulative frequency periodograms have provided useful information to characterize meat products [8,10]. In concrete terms, FFT25 would correspond to the minimum frequency value for which 25% of the total energy would have been received. That is to say, if the 25th percentile of the cumulative frequency were located at x Hz for a particular inspection, this would indicate that 25% of the received signals would have frequencies lower than x Hz—identically for FFT99. For illustrative purposes, Supplementary Material Figure S1 exhibits the cumulative frequency periodogram generated from the FFTs presented in Figure 5.

Finally, the attenuation parameters were also considered in two different ways of calculation. Firstly, given that the nature of the beef loins makes it impossible to distinguish reflections in direction 1, a new procedure has been developed based on the signal’s progressive energy loss [9,10]. This loss is quantified in terms of the time taken to receive the energy, expressed as a multiple of the time of flight TOF (n·TOF), specifically for 20%, 40%, and 60% of the total energy received (denoted as *AT20*, *AT40*, and *AT60*, respectively). By way of an explanatory example, a value of *AT60* equal to 1.03 would indicate that 60% of the total energy has been received in a time equal to the first 1.03 times the TOF. In this way, the greater the value of n·TOF at the ATxx time, the less attenuative the corresponding sample will be. For illustrative purposes, Supplementary Material Figure S2 exhibits the attenuation curve corresponding to the A-scan shown in Figure 4a.

Within the A-scans displaying visible echoes, particularly in direction 2, the attenuation was taken using Equation (1):

(1) $$\alpha =\frac{1}{2d}\ln\left(\frac{A_{i}}{A_{j}}\right)$$

where A*i* and A*j* represent the peak-to-peak amplitudes of echoes *i* and *j*, respectively, and 2*d* corresponds to the distance covered by the ultrasound wave between these two echoes. The evaluation of *α* was based on the analysis of echoes obtained in the A-scan (two in the case of A114 and one in the case of V318). The calculation of α involved plotting ln(A*i*/A*j*) against 2*d*, and the slope of this plot provided the value of the attenuation coefficient.

### 2.4. Data Analysis

Mean values and standard deviations were compiled and analyzed using the XLSTAT 2.1. software package (Addinsoft Pearson Edition 2019, Paris, France). Likewise, linear correlation analysis based on the Pearson correlation coefficient (R) was carried out among physicochemical and ultrasound parameters following the procedures from previous contributions [9]. Correlation coefficients were calculated from the acoustic parameters obtained with the V318 and A114 transducers (velocity (UPV and UPVE), attenuation (AT20, AT40, AT60 and α), and frequency parameters (FFT25 and FFT99)) in both directions, and with the different physicochemical determinations (aW, moisture, lipids, instrumental color, and texture parameters).

## 3. Results and Discussion

### 3.1. Physicochemical Results and Ultrasound Inspection Results

Table 1 displays the mean values and standard deviation of physicochemical parameters. The results were similar to those found in previous studies of beef loin characterization for water activity [12], instrumental color [25], texture profile [26,27], and moisture and lipid content [28]. These results differ greatly from those obtained in pork loin in previous works, particularly in fat and water content [9].

Regarding acoustic characterization, Table 2 shows the results of ultrasound inspection parameters with V318 and A114 transducers. The velocity results range from 1528 to 1567 m/s, depending on the method and transducer used and the direction of the assessment. As can be observed, the mean velocity values obtained by both methods for the same pairs of transducers are similar when considering the error margins. However, the velocities obtained with the A114 transducers (1 MHz) are clearly lower than those obtained with the V318 transducers (0.5 MHz). This difference should primarily be attributed to the different frequencies of the transducers. Higher-frequency transducers have a shorter wavelength, making them sensitive to smaller elements or discontinuities that would be invisible to a longer wavelength. Consequently, the ultrasonic waves would take a longer time in the corresponding inspection, resulting in a lower velocity for the inspected loin piece. In any case, the velocity values are quite similar to those determined in other studies in the literature, with variations due to different measurement conditions (temperature, muscle, inspection technique, inspection frequency). Fariña et al. (2023) [12] reported a velocity of 1584 m/s using 1 MHz contact transducers, showing similar behavior in comparison with the results of the present study. Likely, the small differences may be attributed to the different inspection temperature (4 °C). In comparison with the non-contact methodology, the velocity mean values were higher [12], particularly 1622 m/s, with 0.3 MHz central frequency transducers. The variations in this case are probably explained by the differences in thickness of the samples, as well as the nominal frequencies transducers and methodologies used in the ultrasonic inspection. Other authors estimate the velocity in a range of 1605–1635 [16], but probably this increment is due to the aging process of the samples of this study. Furthermore, in this study, 10 MHz transducers were used. Regarding the comparison with other meat matrices, previous studies stated the UPV on fresh pork loin in a range between 1491 m/s and 1563 m/s [9] in trough-transmission contact methodology. On the other hand, using non-contact transducers determined the velocity in a range between 1587 m/s and 1668 m/s in pork burger patties and considering different temperatures of measurements [11]. With regard to the results presented for the parameters related to frequency and attenuation, they are not comparable to other studies because these parameters were generally not considered, or when they were, the transducers and inspection geometries had different characteristics [14,29,30], which prevents comparison with the current results.

With regard to frequency parameters, first, it should be noted that lower values were found for the V318 transducers compared to the A114, undoubtedly due to the lower central frequency of the former compared to the latter. Not as evident but noteworthy are the results of *FFT25*, and *FFT99* in that their values in direction 1 are lower than in direction 2. This result is a consequence of the greater thickness of the inspections carried out in direction 1 compared to direction 2. Since a greater thickness entails a longer transit path for ultrasonic waves through the sample, the attenuation will also be higher, especially at high frequencies (or shorter wavelengths). This is the reason that explains, for instance, the lower values of*AT20*, *AT40*, and *AT60* in the inspections conducted with the A114 transducers compared to those performed with the V318, or equivalently, the higher values of alpha in the tests with the A114. Higher inspection frequency leads to greater attenuation. For *α* values, this relationship is directly evident in our study. As for *ATxx*, it should be noted that since attenuation parameters are reported as a function of *TOF*, the higher their value (i.e., a longer time to reach a certain percentage of energy), the lower the attenuation, as indeed observed in the aforementioned inspections with the A114.

### 3.2. Correlations between Ultrasonic Parameters and Physicochemical Properties

**Figure 6:**
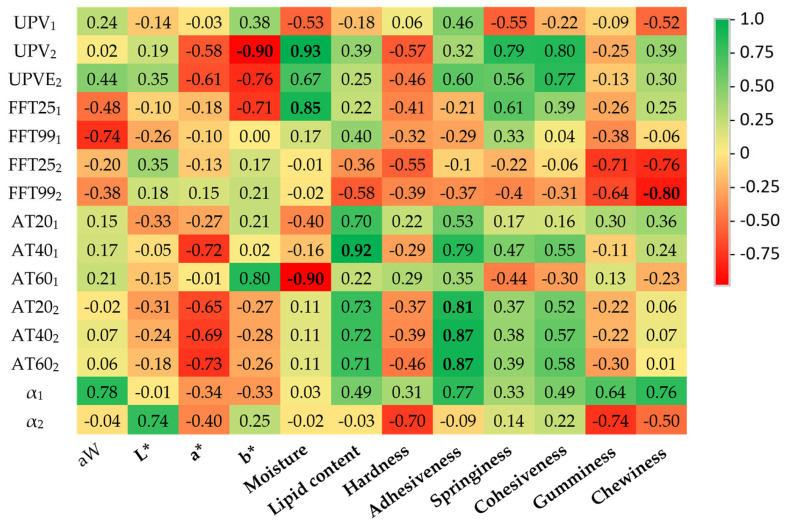
Heatmap of linear correlation matrix (coefficients R) between the V318 transducer acoustic and the physicochemical parameters. UPV = ultrasound pulse velocity (m/s); UPVE = ultrasound pulse velocity obtained from visible echoes (m/s); FFT25/FFT99 = fast Fourier transform corresponding to the 25th and 99th percentiles; AT20/AT40/AT60 = attenuation corresponding to reaching 20%, 40%, and 60% of the energy; α = attenuation obtained as energy lost; aW = water activity; L* = lightness; a* = redness; b* = yellowness. Sub-index numbers refer to the directions of the measurements. Bold numbers mean significant correlations.

Figure 6 shows the Pearson linear correlation coefficients (R) observed in this correlation study. Several significant correlations (both positive and negative) were found between the ultrasound parameters determined using the V318 transducers and the physicochemical results. It is striking that the ultrasonic parameters traditionally used in meat quality studies (UPV and α) exhibit scarcely any significant relationships with any of the studied physicochemical parameters. In contrast, the less common acoustic parameters related to frequency and attenuation do manage to correlate significantly with the quality parameters of beef loins. Thus, the positive correlations identified between fat content and attenuations were notably significant in the case of AT40 (direction 1). This outcome implies reduced attenuation experienced by ultrasonic waves in samples with higher fat infiltration. We could attribute this phenomenon to the coupling effect exerted by the infiltrated fat, which promotes energy transmission as it propagates through the meat specimen. Other authors have emphasized the relationship between ultrasonic parameters and fat content [20,31,32], so these correlations are in line with other studies on beef loin based on ultrasonic inspection. In the same vein, we could explain the negative correlation coefficients found between moisture and AT60 in direction 1. Once more, this would suggest a greater challenge in the transmission of acoustic energy in those samples with a higher water content. Moisture also exhibits a positive correlation with velocity (UPV_2_) and frequency components (FFT25_1_). These results would imply that the ultrasonic signal passes through samples with higher water content more rapidly and that lower frequencies are more significantly attenuated proportionally. These findings confirm the sensitivity of ultrasonic parameters in assessing water content, which has already been observed in other meat products [8,20].

As for the physicochemical parameters related to color, only b* exhibited significant correlation with velocity (UPV_2_). The lack of mechanical significance for the b* parameter makes it challenging to provide a rationale in favor of or against such a correlation.

As for the textural parameters, adhesiveness exhibits the most significant correlation coefficients with the ultrasonic parameters, particularly with attenuation. This parameter indicates the effort required to separate the food surface from another surface. In our study, this result suggests that the samples with less attenuation of ultrasonic waves are the ones with higher adhesiveness values. Also noteworthy is the negative relationship of the frequency components, particularly significant in the case of FFT99_2_, with chewiness, which is related to the number of chews required to render a solid product to the necessary condition for swallowing. Therefore, tougher loins (harder meat) would be those that proportionally attenuate higher frequencies more.

**Figure 7:**
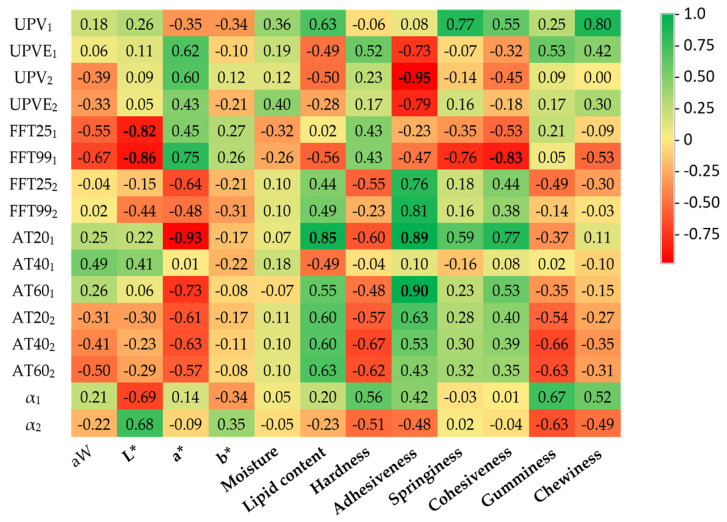
Linear correlation coefficients (R) between the A114 transducer acoustics parameters and the physicochemical parameters. UPV = ultrasound pulse velocity (m/s); UPVE = ultrasound pulse velocity obtained from visible echoes (m/s); FFT25/FFT99 = fast Fourier transform corresponding to the 25th and 99th percentiles; AT20/AT40/AT60 = attenuation corresponding to reaching 20%, 40%, and 60% of the energy; α = attenuation obtained as energy lost; aW = water activity; L* = lightness; a* = redness; b* = yellowness. Sub-index numbers refer to the directions of the measurements. Bold numbers mean significant correlations.

Figure 7 displays the correlation coefficients among physicochemical and ultrasonic parameters using the A114 transducers. As observed, this pair of transducers exhibited less significant correlation coefficients in comparison to the V318 transducers. Of course, now none of the traditional ultrasonic parameters (UPV and α) appear to be particularly sensitive to any of the traditionally indicative physicochemical parameters of meat quality either. Overall, the most significant coefficients reaffirm the results presented with the V318 transducers. For example, this is the case with fat content and ultrasonic attenuation, once again demonstrating that the samples with lower sound attenuation are those with a higher amount of infiltrated fat. Similarly, the relationship between adhesiveness and attenuation follows the same pattern, with samples exhibiting lower ultrasonic attenuation being more adherent. In the case of A114, another parameter needs to be considered: cohesiveness in relation to the frequency components, which is particularly significant in the case of FFT99_1_. Thus, this relationship would indicate that meat samples that withstand a high degree of deformation before breaking are the ones that proportionally attenuate high frequencies the most. In this manner, it only remains to comment on the negative coefficients found between certain ultrasonic parameters linked to frequency (FFT25_1_ and FFT99_1_) and attenuation (AT20_1_) with color parameters (L* and a*). Therefore, even though color always serves as an indicator of the water and fat content of the meat, the absence of mechanical equivalence for these variables makes the assessment of these results complex.

As can be observed, non-destructive ultrasonic inspection of identical meat samples has yielded identical results in some cases, but also others in which specific correlations appear only with certain pairs of transducers. This phenomenon can be attributed initially to both the ultrasonic inspection frequency and the inherent characteristics of the meat samples. It is well known that the choice of ultrasonic inspection frequency plays a crucial role in the variability of results. Different frequencies have the capability to penetrate meat at various depths and as a result interact with different internal features and structures of the samples. Lower frequencies tend to penetrate more deeply and can reveal information about the overall texture and density of the meat, while higher frequencies focus on superficial features, such as the presence of muscle fibers and connective tissue. Thus, the variability in results can be explained by the sensitivity of ultrasonic frequency to different meat characteristics [33,34]. Furthermore, it is important to consider the heterogeneity in meat samples, not only at a microstructural level but also, for instance, in terms of the size and orientation of muscle fibers and infiltrated fat. This impacts the uniformity of ultrasonic measurements and can account for the observed differences [31,32].

## 4. Conclusions

The conducted studies postulate the ultrasonic inspection as a promising non-destructive tool for characterizing beef loin. Specifically, the results obtained with transducers of 500 kHz and 1 MHz demonstrate that the commonly studied ultrasonic parameters in food inspection in general and meat inspection in particular—namely, velocity and attenuation—do not provide an effective means of acoustically assessing the beef loins under study. Instead, other more innovative parameters, such as those linked to the frequency components present in the received signal or the attenuations experienced by the waves in terms of the time required to receive a certain percentage of energy, do exhibit significant correlations with textural and physicochemical parameters that are traditionally indicative of meat quality.

In particular, studies conducted with transducers at both frequencies conclude that there are several positive correlations between fat content and attenuations. This result implies reduced attenuation experienced by ultrasonic waves in samples with higher fat infiltration. This phenomenon can be attributed to the coupling effect exerted by infiltrated fat, which promotes energy transmission as it propagates through the meat specimen. Similarly, adhesiveness is the textural parameter that exhibits the most significant correlation coefficients with ultrasonic parameters, particularly with attenuation. This result suggests that samples with lower ultrasonic wave attenuation have higher adhesiveness values.

Other meat textural parameters only show significant correlations with the ultrasonic measurements at one of the two frequencies. Thus, inspections carried out with 500 kHz transducers also indicate a relationship between frequency components and chewiness, with tougher loins (harder meat) proportionally attenuating higher frequencies. On the other hand, inspections conducted with 1 MHz transducers demonstrate a close relationship between cohesiveness and frequency components, indicating that meat samples capable of withstanding a high degree of deformation before breaking proportionally attenuate higher frequencies.

Future work should assess and confirm the suitability of ultrasonic inspection frequency based on the physicochemical parameter to be evaluated. In this regard, the variability in the results of non-destructive ultrasonic inspection of meat samples appears to be related to the complex interaction between ultrasonic inspection frequency and the specific characteristics of the samples.

## Figures and Tables

**Table 1:** Mean values and standard deviation of physicochemical parameters of beef loin samples.

Parameters	Mean Values ± Standard Deviation
aW (Water activity)	0.974 ± 0.003
Lightness (L*)	41.30 ± 3.52
Redness (a*)	14.11 ± 1.67
Yellowness (b*)	5.44 ± 1.76
Moisture (%)	72.67 ± 2.22
Lipids g/100 (%)	3.10 ± 0.03
Hardness (g)	1757 ± 403
Adhesiveness (g·s)	−79.61 ± 34.12
Springiness (g)	0.45 ± 0.07
Cohesiveness	0.45 ± 0.07
Gumminess (g)	770 ± 121
Chewiness (g)	343 ± 76

**Table 2:** Acoustic parameters obtained with the V318-SU and A114-S transducers (mean values ± standard deviation).

	V318		A114	
	Direction 1	Direction 2	Direction 1	Direction 2
UPV (m/s)	1567 ± 7	1565 ± 15	1548 ± 6	1528 ± 11
UPVE (m/s)	nv	1558 ± 7	nv	1532 ± 8
FFT25 (Hz)	354,017 ± 39,995	389,803 ± 11214	702,593 ± 1781	742,577 ± 1781
FFT99 (Hz)	680,115 ± 19,385	723,840 ± 11845	1,066,764 ± 4953	1,179,975 ± 4953
AT20	1.020 ± 0.002	1.021 ± 0.002	1.017 ± 0.001	1.018 ± 0.001
AT40	1.023 ± 0.004	1.030 ± 0.003	1.019 ± 0.001	1.023 ± 0.001
AT60	1.030 ± 0.008	1.033 ± 0.003	1.022 ± 0.001	1.026 ± 0.002
α (np/m)	184 ± 13	197 ± 20	237 ± 4	249 ± 13

UPV = ultrasound pulse velocity; UPVE = ultrasound pulse velocity obtained from visible echoes; FFT25/FFT99 = frequency values from fast Fourier transform corresponding to the 25th and 99th percentiles; AT20/AT40/AT60/= attenuation parameters corresponding to reaching 20%, 40%, and 60% of the energy; α = attenuation coefficient; nv = means non-visible echoes.

## Data Availability

The datasets used and/or analyzed during the current study are available from the corresponding author on reasonable request.

## Supplementary Materials

The following supporting information can be downloaded at https://www.mdpi.com/article/10.3390/s23239564/s1. Table S1: Characteristics of the transducer models used; Figure S1: Cumulative frequency periodogram corresponding to the FFT shown in Figure 5. The 25th and 99th percentiles of the frequencies are explicitly indicated; Figure S2: Attenuation curve corresponding to the A-scan shown in Figure 4a. The AT20, AT40, and AT60 attenuation values are explicitly indicated.
