# Supplementary material for: Acoustic Characterization Study of Beef Loins Using Ultrasonic Transducers
Source: Sensors (Basel). 2023 Dec 1;23(23):9564. doi: 10.3390/s23239564 (PMC10708575; doi:10.3390/s23239564)
Supplement: Supplementary file 1 [file sensors-23-09564-s001.zip › sensors-2700848-supplementary.pdf]

Table S1. Characteristics of the transducer models used.

Model	Diameter (cm)	Frequency (kHz)	Wavelength (mm)	-6 dB bandwidth (%)	N (cm)	φ (°)	Waveform duration (μs)		
							14dB	20 dB	40 dB
Panametrics V318-SU	1.9	500	3.14	61.93	2.87	11.6	3.680	4.888	16.600
Panametrics A114S	1.9	1000	1.54	64.59	5.86	5.67	2.540	2.900	6.079

UPV=1570 m/s in case of V318 and 1540 m/s in case of A114 were taken for the calculation of wavelength, N, and φ
N= near-field length
φ= beam angle
The bandwidth and waveform direction values were obtained according to the following conditions:
Damping: 500 ohms; Attn: 36 dB; Gain: 40 dB

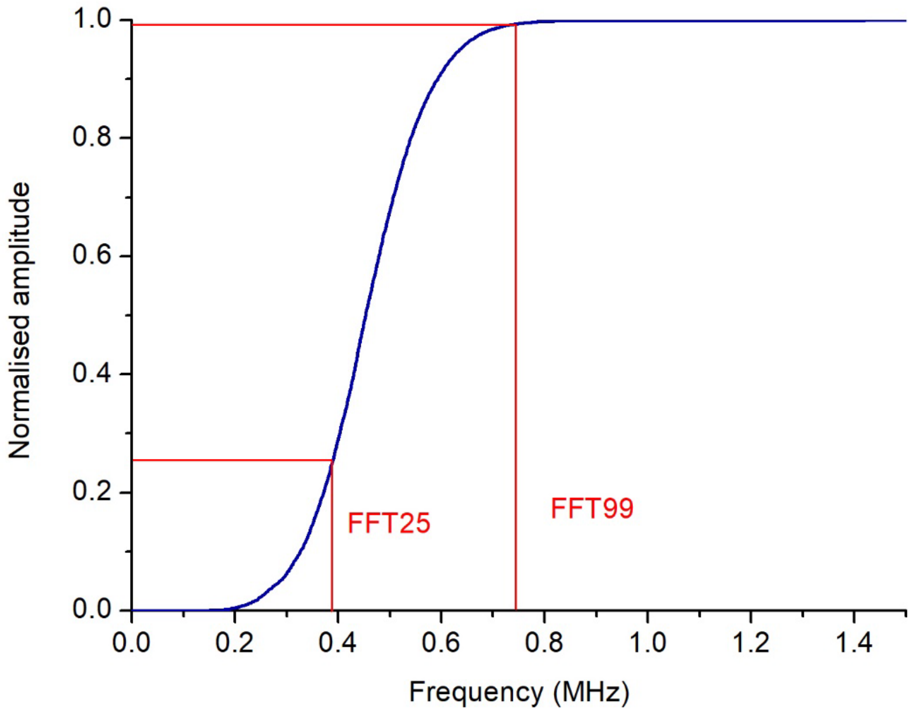

Figure S1. Cumulative frequency periodogram corresponding to the FFT shown in Figure 5. The 25th and 99th percentiles of the frequencies are explicitly indicated.

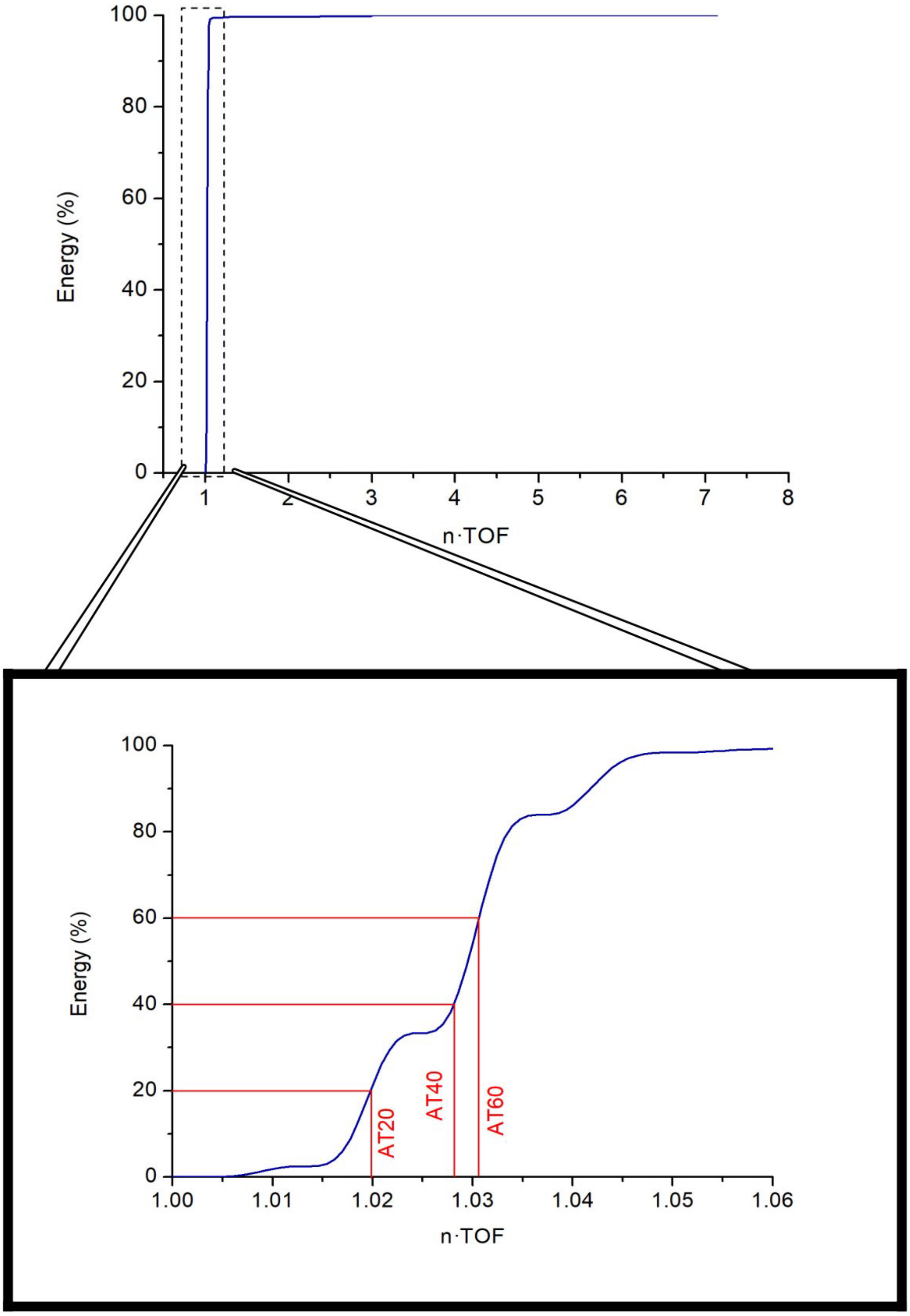

Figure S2. Attenuation curve corresponding to the A-scan shown in Figure 4a. The AT20, AT40, and AT60 attenuation values are explicitly indicated.
